# Maternal dietary diversity and micronutrient adequacy during pregnancy and related factors in East Gojjam Zone, Northwest Ethiopia, 2016

**DOI:** 10.1186/s12884-019-2299-2

**Published:** 2019-05-15

**Authors:** Tebikew Yeneabat, Haweni Adugna, Tarekegn Asmamaw, Muluken Wubetu, Melaku Admas, Getachew Hailu, Asres Bedaso, Tadele Amare

**Affiliations:** 1 Department of Midwifery, Health Science College, Debre Markos University, Debre Markos, Ethiopia; 2 Department of Nursing and Midwifery, School of Allied Health Sciences, Addis Ababa University, Addis Ababa, Ethiopia; 3 Mariestpes International Ethiopia, Regional Advisor, Addis Ababa, Ethiopia; 4 Department of Pharmacy, College of Health Sciences, Debre Markos University, Debre Markos, Ethiopia; 5 School of Public Health, College of Medicine and Health Sciences, Bahir Dar University, Bahir Dar, Ethiopia; 6 School of Nursing and Midwifery, College of Medicine and Health Sciences, Hawassa University, Hawassa, Ethiopia; 7 Department of Psychiatry, College of Medicine and Health Sciences, University of Gondar, Gondar, Ethiopia

**Keywords:** Community based, Dietary diversity, Pregnant women, Micronutrient adequacy

## Abstract

**Background:**

Monotonous and less diversified diets are associated with micronutrient deficiency. Evidence on maternal dietary intakes during pregnancy is essential to achieve the 2025 global nutrition target and reduce maternal and child mortalities. This study assessed pregnant women’s dietary diversity and identified factors associated with inadequate dietary diversity in East Gojjam Zone.

**Methods:**

We conducted a community-based cross-sectional study between April and June 2016. Eight hundred thirty-four pregnant women were randomly sampled. The Women Dietary Diversity Score tool developed by the Food and Agricultural Organization (FAO) and Food and Nutrition Technical Assistance (FANTA) was used. Data were entered into EpiData with double entry verification, and analysis was done using IBM SPSS version 20. Level of significance was set to *P* < 0.05 with 95% confidence interval (CI) to identify the independent factors associated with inadequate dietary diversity.

**Results:**

The mean (±SD) dietary diversity score was 3.68 (±2.10). Inadequate dietary diversity was prevalent in 55% [95% CI (52.3–59.3%)] of pregnant women, or indirectly micronutrient was inadequate in more than half of the pregnant women. Commonly consumed dietary groups were legumes, nuts, and seeds (85.5%) followed by starchy staples (64.7%). Inadequate dietary diversity was higher among non-educated [Adjusted Odds Ratio (AOR) = 7.30, 95% CI (2.35–22.68)] compared to college and above completed women. Wealth index had significant association with dietary diversity, in which women in the poorest [AOR = 8.83, 95% CI, (1.60–48.61)], poorer [AOR = 6.34, 95% CI (1.16–34.65)], poor [AOR = 8.46, 95% CI (1.56–45.70)], and richer [AOR = 6.57, 95% CI (2.16–20.01)] had higher odds of inadequate dietary diversity. Those who had not received dietary counseling had three folds [AOR = 3.31, 95% CI (1.49–7.35)] of inadequate dietary diversity compared to their counterparts. Less likelihood of inadequate dietary diversity was among women with an increased meal frequency [AOR = 0.53, 95% CI (0.38–0.74)].

**Conclusion:**

Consumption of less diversified food during pregnancy is common in the study area. Adequacy of micronutrients is insufficient for more than half of the studied pregnant women. We conclude that being non-educated affects pregnant women to depend on less diversified diet. Providing dietary counseling during pregnancy can improve nutritional practice for pregnant women.

## Background

Women dietary diversity score (WDDS) is the number of different food groups consumed by each woman over a given reference period [1–4]. It is a qualitative measure of food consumption, used as a proxy of micronutrient adequacy at an individual level [5]. Unlike the household dietary diversity score (HDDS), it considers foods consumed outside of the home in a given reference period, in the last 24 h. Dietary diversity is the most essential element to prevent micronutrient deficiency. It reflects the concept that increasing the variety of foods and food groups in the diet helps to ensure the adequate intake of essential nutrients and so promotes good health [3].

Maternal undernutrition is a global burden and is still the neglected health problem particularly in the developing countries where maternal mortality, low birth weight, and childhood stunting are the major health problems [6]. It is commonly due to micronutrient deficiencies that occur when people do not have access to micronutrient-rich foods such as fruit, vegetables, animal products, and fortified foods. The most vulnerable groups are pregnant and lactating women and young children [7, 8]. According to the evidence from a large epidemiological study, more than one-third of child deaths and 11% of the total disease burden worldwide are due to maternal and child undernutrition [9].

A World Bank report states that the impact of poor nutrition on maternal and child health is lasting and its consequences are reaching far beyond health. Poor nutrition’s impact on maternal and child health has the potential to reduce the economic output of countries by 2–3% annually [10, 11]. Almost all (99%) of maternal deaths annually occur in developing countries [7], where many of the cases have a link with poor nutrition. For example, iron deficiency anemia is the most common cause of indirect maternal deaths in which pregnant women with iron deficiency are at risk of death, hemorrhage, and sepsis during childbirth [7, 9, 12, 13].

The problem of micronutrient deficiency is a double burden in pregnant women that leads to poor fetal development and a higher risk of pregnancy complications [14]. Because of pregnancy is characterized by increased demand for varieties of micronutrients like iodine [15] by the feto-placenta as well as the change in metabolism [16], the majority of serum micronutrients are low during pregnancy which becomes severe as gestation progress [17]. As a result, it has both short- and long-lasting effects on intra-uterine fetal development, intrapartum problems like obstructed labor, neonatal and childhood health problems and affects the quality of maternal life during pregnancy and postnatal period [6, 18–27]. Recently, the agenda of maternal and child health has been given higher emphasis in Ethiopia; and nutrition interventions like national nutrition program (NNP) are among the possible means to avert the problem; and pregnant and lactating women, are the target groups of NNP of the Ministry of Health of Ethiopia [28].

In the year 2025, the WHO has planned to reduce anemia by 50% and low birth-weight by 30% [29] which needs evidence on the underlying dietary profile of the pregnant women which is vital to design appropriate intervention strategies helping to avoid preventable maternal morbidities and mortalities accounted by nutritional problem [30]. Data on women dietary intakes and micronutrient adequacy are urgently needed to help characterize the magnitude and distribution of the problem, to mobilize resources to address it, and to design effectively, target, monitor, and evaluate actions aimed at reducing the burden of women’s micronutrient malnutrition in the short and medium term. There has been no previous study conducted in Amhara Region on dietary diversity among pregnant women. Therefore, this study was aimed to assess pregnant women dietary diversity and micronutrient adequacy and identify factors associated with inadequate dietary diversity in East Gojjam Zone.

## Methods

A community-based cross-sectional study was done in East Gojjam Zone. Quantitative data were collected to study women dietary diversity. It was done between April and June 2016. This period was preferred because the time is neither harvest nor food shortage as the majority of the population in the study area are engaged in agriculture. According to the 2015 estimate, the number of women in the reproductive age group is 605,936. According to the 2014 Demographic and Health Survey mini report [31], the proportion of pregnant women in the Amhara region is 6.2%. Using the conversion rate, the estimated number of pregnant women in the study area is 37,568. All pregnant women in their reproductive age group in the study area were the source population for this study.

### Sample size determination and sampling procedure

The sample size was calculated using a single population proportion formula. The proportion of pregnant women who took adequate diet was 44% [32].

$$ n=\frac{\boldsymbol{z}\ {\frac{\boldsymbol{\alpha}}{\mathbf{2}}}^{\mathbf{2}}\boldsymbol{p}\left(\mathbf{1}-\boldsymbol{p}\right)\ }{{\boldsymbol{d}}^{\mathbf{2}}} $$

$$ n=\frac{\mathbf{1.9}{\mathbf{6}}^{\mathbf{2}}\boldsymbol{x}\ \mathbf{0.44}\left(\mathbf{1}-\mathbf{0.44}\right)\ }{{\mathbf{0.05}}^{\mathbf{2}}}=\mathbf{378.628} $$

Then the calculated sample size was multiplied by the *Design effect* of 2 because a multistage sampling was used. Anticipated 10% of non-response rate was added and the final sample size was 834.

**Fig. 1:**
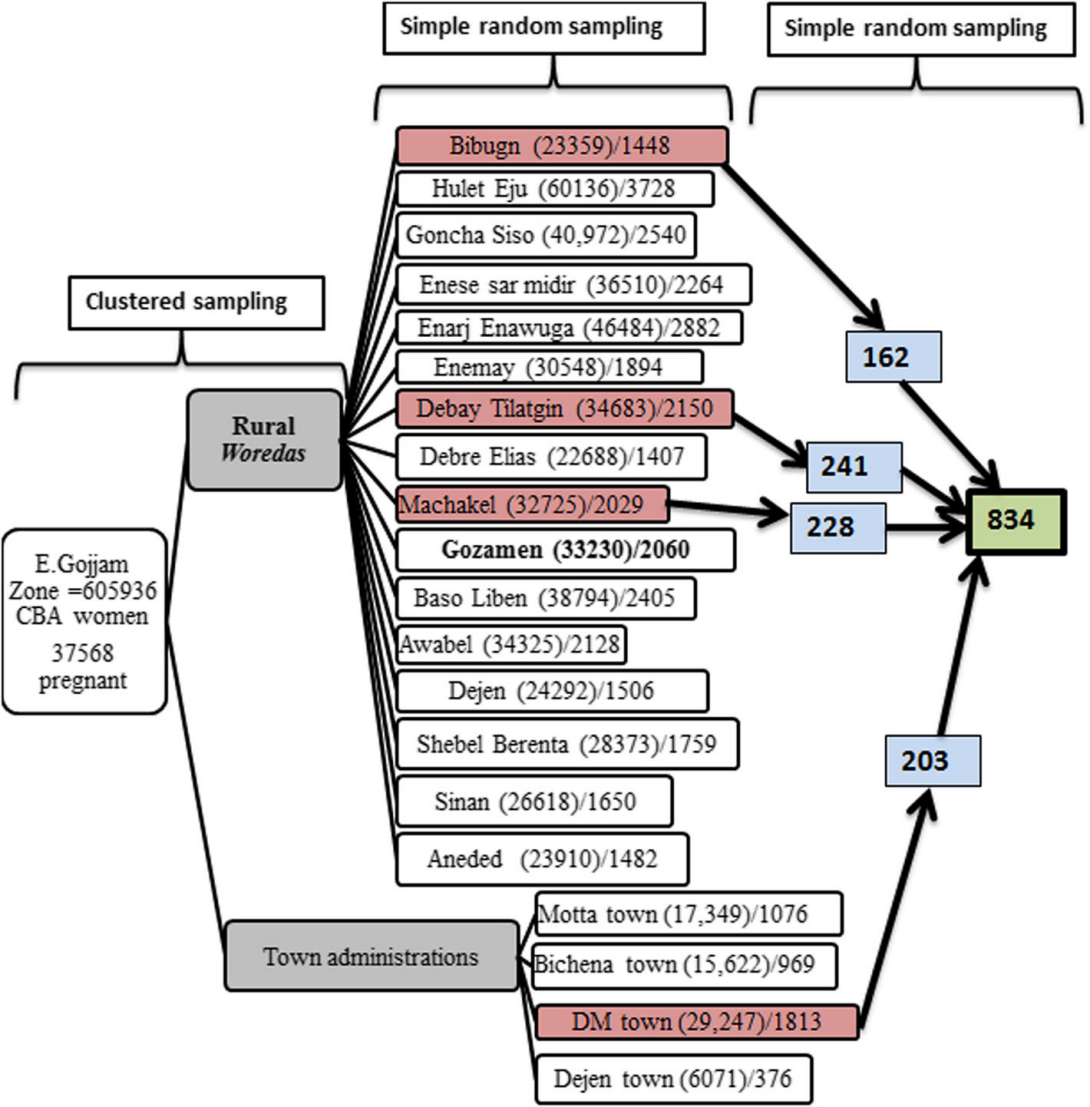
Schematic presentation of sampling techniques, East Gojjam, 2016. (*Note: The number in the brackets indicate total reproductive age group women in the woreda, and the number after solidus (/) sign is pregnant women in the woreda*)

Multistage sampling technique was used to select the interviewee. East Gojjam Zone was clustered into ‘woredas’ (In Ethiopia, ‘*Woreda’* is the administrative district that further divided into smaller administrative areas called ‘*Kebele’*). There are 20 woredas (16 rural and 4 urban *woredas*). Four woredas (three from rural and one from urban) were selected randomly. Proportional allocation to sample size was done, and respondents were selected randomly using the registration book obtained from health posts in the selected Woredas (Fig. 1).

### Inclusion and exclusion criteria

Pregnant women who have attained the full age of 18 years, and had registered in the family folder by health extension workers were included. Those who had been registered in the family folder and had residency relocation out of the selected *woredas* were excluded.

### Data quality and processing

Individual’s dietary diversity score measurement tool developed by the Food and Agricultural Organization (FAO) and the Food and Nutrition Technical Assistance FANTA [3, 4] was used. Other socio-demographic questionnaires were used from literature [33–35].

The status of food security was assessed by using the locally validated household food insecurity questionnaire that contained six items [34]. The respondents were asked for any worry on food shortage in the last 12 months rated as 1 for Yes and 0 for No. A response of ‘***Yes’*** for any of the six questions were reported as food insecurity in this research. Household economic status was assessed using the tool adopted from the Ethiopian Demographic and Health Survey (EDHS) 2011 [33] which asks whether the household has fixed assets or not. The WDDS tool contains 10 food groups. The tool is a dichotomous indicator of whether or not pregnant women have consumed at least five out of 10 food groups previous day or night, that is 24 h recall [4]. We modified the tool into the local context based on the food lists to reflect locally available foods, and translation was done Amharic, the local language in the study area. Moreover, Human nutrition experts (Holders of master’s degree in Human nutrition, and working at Debre Markos University Health Science College as academic staffs) were consulted to improve the local validation of the tool. Before the commencement of the study, a detail of the methods (the tool, data collection technique, ethical issues, data quality, data processing, and analysis) was presented to research experts of Debre Markos University College of Health Sciences. All the comments and questions raised by the research experts were acknowledged, and we have considered for improving the validity of the study. Once the translation and adaptation of the questionnaire were completed, interviewers were trained for 3 days to conduct the interviews. Training includes classroom instruction, discussion, and field practice. Interviewers were degree holders in the field of Nursing, four in number and recruited to conduct the interview based on their previous experience of data collection. We pretested the tool in 5% of the study respondents (42 women) in West Gojjam Zone. Internal consistency of the tool was identified (Cronbach’s alpha: 0.73 for women Dietary Diversity Score tool, and 0.93 for Household food security. Collected data were checked a daily before further data processing. Pregnant women were interviewed to respond on all foods that they had eaten inside or outside the home, irrespective of where they were prepared. EpiData [36] was used to enter the data with double entry verification to avoid data entry errors. Finally, data were exported to IBM SPSS version 20 [37] for analysis.

### Variables

Women Dietary diversity Score categorized as adequate (consumption of at least five among 10 food groups) and inadequate (consumption of less than five food groups) [4] was an outcome variable. Individual’s socio-demographic characteristics, obstetric and gynecologic characteristics and level of household food security were independent variables.

### Data analysis

IBM SPSS version 20 software was used for data analysis. A logistic regression model was used to identify independent factors of inadequate WDDS. Adequacy of food intake was considered for those women who ate at least five food groups out of 10 food groups [4]. The score below five was inadequate, and the score equal to five or more was considered as adequate. Finally, women dietary diversity was dummied as 1 = adequate dietary diversity and 0 = inadequate dietary diversity. Wealth index in quintile was constructed using the principal component analysis. Frequency distribution for categorical variables, and mean with standard deviation for continuous variables were computed to describe the variables. Model building was executed in two stages. Firstly, each independent variable was entered into the bivariable logistic regression model to see its association with dietary diversity. Variables with missing values were not included in the model. The odds ratio was determined and *p* < 0.05 with 95% CI was used to ascertain statistical significance. Secondly, independent variables with *p* < 0.2 in the bivariable logistic regression were included in the multivariable logistic regression model to identify the independent variables associated with dietary diversity.

Model fitness was checked using Hosmer and Lemeshow statistic test at *p* > 0.05.

## Results

Of the total 834 pregnant women sampled, data from 759 respondents were included in the analysis giving the response rate 91%. Of these, 37.2% were from urban, and 62.8% were from rural. The data collected from 75 respondents were not included in the analysis due to incomplete response.

The mean (±SD) age of the respondents was 29.06 (±5.44) years. Majority of the respondents were from Orthodox Tewahido Christian religion, and almost all (99.87%) were Amhara in their ethnicity (Table 1).

**Table 1:** Socio-demographic characteristics of study population, East Gojjam Zone, Northwest Ethiopia, 2016

Variables	Frequency	Percent (%)
Age of the respondents in years		
< = 19	3	0.4
20–24	162	21.3
25–29	282	37.2
30–34	159	20.9
45–39	116	15.3
> =40	37	4.9
Religion		
Orthodox Tewahido	755	99.47
Other (Muslims and protestant)	4	0.53
Educational status		
Illiterate	383	50.46
Primary school	150	19.76
Secondary school	91	11.99
Diploma and above	135	17.79
Marital status		
Married	731	96.31
Widowed	6	0.79
Divorced	16	2.11
Unmarried	6	0.79
Occupation		
Salary based employed	67	8.83
Non-salary employed	692	91.17
Place of residence		
Rural	477	62.85
Urban	282	37.15
Wealth index		
The poorest	151	19.9
Poorer	152	20.0
Poor	152	20.0
Richer	200	26.4
The richest	104	13.7

The majority, 484(63.8%) of the interviewed pregnant women had one to three history of lifetime pregnancy. Nearly one-fifth (18.2%) of pregnant women had had no antenatal care for the recent pregnancy until the date of the interview. Among those who had visited ANC: 20.7% had four or more visits, and 13.8% had one ANC visit. About 90% of pregnant women reported that they had received counseling on dietary intake during their ANC visit (Table 2).

**Table 2:** Obstetric characteristics of pregnant women in East Gojjam Zone, Northwest Ethiopia, 2016

Variables	Frequency	Percent (%)
Lifetime pregnancy		
1–3	484	63.8
> 3	275	36.2
ANC received for current pregnancy		
Yes	621	81.8
No	138	18.2
Counseling of dietary intake		
Yes	557	89.7
No	64	10.3
Lifetime live birth		
Zero	213	28.1
One	149	19.6
2–4	308	40.6
Five and more	89	11.7
Number of alive children		
Zero	223	29.4
One	153	20.2
Two and more	313	50.4

## Factors associated with dietary diversity

The mean dietary diversity (±SD) score in the last 24 h of data collection period was 3.68 (±2.10). Nearly one in seven (14%) pregnant woman consume a monotonous diet. The prevalence of inadequate dietary diversity was 55.7% [95% CI (52.3–59.3%)]. Commonly consumed food groups were legumes, nuts and seeds (85.5%) followed by starchy staples (64.7%).

The bivariable logistic regression model showed that all variables included in the analysis were found significantly associated with dietary diversity of pregnant women in the study area. Marital status and the status of antenatal care utilization were excluded from logistic regression models due to a few observations concerning the outcome variable and higher standard errors.

Maternal age was significantly associated with WDDS in bivariable logistic regression [Crude Odds Ratio (COR) = 1.05, 95% CI (1.02–1.08)] that revealed as age increases by a year, the likelihood of inadequate dietary diversity increases by 0.05, however, statistical significance was not observed in multivariable logistic regression model [Adjusted Odds Ratio (AOR) = 1.02, 95% CI (0.96–1.109)].

Only non-educated pregnant women had significantly inadequate dietary diversity compared to college and above completed pregnant women. Non-educated pregnant women were about 7.3 [AOR = 7.3, 95% CI (2.35–22.68)] times more likely to have inadequate dietary diversity compared to those who completed college and above educational level. Primary and secondary school educational level had no significant association with dietary diversity in multivariable logistic regression. A significant association between WDDS and partner’s educational status was observed in bivariable logistic regression but insignificant in multivariable logistic regression.

Women’s occupational status was significantly associated with WDDS in bivariable logistic regression. Women engaged in a non-salary based occupation were about three times more likely to had inadequate dietary diversity score compared to those engaged in salary based occupation. The association was not observed in the multivariable logistic regression model [AOR = 1.06, 95% CI (0.82–1.32)]. Similarly, place of residence (Rural versus Urban) was not associated with WDDS in multivariable logistic regression [AOR = 2.57, 95% CI (0.81–8.20)].

Economic status, measured in terms of the wealth index, also was assessed for possible contribution to the dietary diversity of pregnant women. Of the categories of the wealth index, classified in quintile, women with wealth index of the poorest [AOR = 8.83, 95% CI (1.60–48.61)], poorer [AOR = 6.34, 95% CI (1.16–34.65)], poor [AOR = 8.46, 95% CI (1.56–45.70)], and richer [AOR = 6.57, 95% CI (2.16–20.01)] were more likely to have inadequate dietary diversity compared to the richest. The status of food insecurity was significantly associated with dietary diversity in the bivariable logistic regression but insignificant in multivariable logistic regression. On the other hand, meal frequency of pregnant women significantly reduced the likelihood of inadequate diet by 49% [AOR = 0.47, 95% CI (0.38–0.74)].

The number of lifetime pregnancy and the number of lifetime live birth were associated with dietary diversity in bivariable but not in multivariable logistic regression. The increased number of ANC visits for the current pregnancy significantly had lower odds of inadequate dietary diversity in bivariable logistic regression but insignificant in multivariable logistic regression. However, receiving dietary counseling during ANC visits was significantly associated with dietary diversity. Those who had not received dietary counseling were more than three times more likely to have inadequate dietary diversity [AOR = 3.31, 95% CI (1.49–7.35)] (Table 3).

**Table 3:** Bivariable and multivariable logistic regression of factors associated with dietary diversity among pregnant women in East Gojjam Zone, Northwest Ethiopia, 2016 (*Model fitness checked with Hosmer and Lemeshow Test (χ*^*2*^ *= 4.798, P = 0.779)*

Variables (*Mean and standard deviation was used for continues variables)*	Dietary diversity					
	Inadequate	Adequate	COR (95% CI)	P	AOR (95% CI)	P
Age	29.06 (± 5.44)		1.05 (1.02–1.08)	0.001	1.02 (0.96–1.09)	0.471
Educational status						
No education	303	80	35.54 (19.07–45.18)	< 0.001	7.30 (2.35–22.68)	0.001
Primary school	82	68	11.32 (5.87–21.81)	< 0.001	2.31 (0.83–6.44)	0.109
Secondary school	25	66	3.56 (1.71–7.41)	0.001	1.253 (0.49–3.19)	0.637
College and above	13	122	1		1	
Educational status of partner						
No education	250	71	25.61 (15.26–42.98)	< 0.001	0.93 (0.31–2.85)	0.907
Primary school	88	42	15.24 (8.55–27.15)	< 0.001	1.20 (0.44–3.26)	0.725
Secondary school	41	57	5.23 (2.87–9.53)	< 0.001	1.69 (0.76–3.72)	0.196
College and above	22	160	1		1	
Woman’s Occupation						
Salary based	22	45	1		1	
Non-salary based	401	291	2.82 (1.66–4.80)	< 0.001	1.06 (0.82–1.37)	0.649
Place of residence						
Rural	366	111	13.02 (9.08–18.66)	< 0.001	2.57 (0.81–8.20)	0.111
Urban	57	225	1			
Number of lifetime pregnancy						
1–3	233	251	1		1	
> 3	190	85	2.41 (1.76–3.29)	< 0.001	0.71 (0.31–1.61)	0.457
Number of lifetime live birth	1.97 (± 1.81)		1.33 (1.22–1.45)	< 0.001	0.85 (0.65–1.10)	0.208
Number of ANC visit	2.67 (± 0.97)		0.75 (0.64–0.89)	0.002	0.92 (0.73–1.15)	0.457
Received dietary counseling						
Yes	257	300	1		1	
No	50	14	4.17 (2.25–7.72)	< 0.001	3.31 (1.49–7.35)	0.003
Wealth index (in quintile)						
The poorest	117	34	86.03 (29.51–250.77)	< 0.001	8.59 (1.56–47.34)	0.014
Poorer	110	42	65.48 (22.67–189.13)	< 0.001	6.34 (1.16–34.65)	0.033
Poor	125	27	115.7 (39.2–341.7)	< 0.001	8.46 (1.56–45.70)	0.013
Richer	67	133	12.59 (4.44–35.69)	< 0.001	6.57 (2.16–20.01)	0.001
The richest	4	100	1		1	
Food insecurity						
Yes	110	42	2.46 (1.67–3.63)	< 0.001	0.73 (0.40–1.33)	0.300
No	313	294	1		1	
Meal frequency	3.63 (± 0.84)		0.26 (0.20–0.32)	< 0.001	0.530 (0.38–0.74)	< 0.001

## Discussion

We were aimed at identifying pregnant women dietary diversity and associated factors in East Gojjam Zone. Although pregnancy is among the joyful events that all childbearing women experience, it is the time when pregnant women have nutritional vulnerabilities due to the physiologic effects of pregnancy demanding additional nutrition [15, 16]. A review article [38] indicated multiple micronutrient supplementations during pregnancy benefits to reduce poor obstetric outcome. However, encouraging pregnant women to consume diversified food from locally available sources is more feasible than micronutrient supplementation in resource-limited countries like Ethiopia where universal health coverage is far to reach. Dietary diversity scores have been positively correlated with increased mean micronutrient adequacy of foods. FAO proposed the new minimum dietary diversity for women consisting of 10 food groups and a dichotomous indicator to indicate minimum dietary diversity when consuming at least five food groups out of 10 [3]. It has been indicated that all women during pregnancy need varieties of diet and micronutrient supplements [39].

The mean dietary diversity score of this study is less compared to the results of the study done in Kenya [40] and Ghana [41]. A nearly similar result has been reported from the Tigray region, Ethiopia [42]. This study shows more than half of the pregnant women’s diet was inadequate. The study done in Pakistan [43] has also reported about half of the studied pregnant women were not consuming adequate food. This result is higher compared to the study done in Kenya [40]. Previous studies confirmed that the prevalence of anemia during pregnancy is more among pregnant women with inadequate dietary intake [44, 45]. This study suggests that more than half of the pregnant women in the study area might be prone to micronutrient deficiencies.

Studies revealed multiple factors are associated with pregnant women dietary diversity score [46, 47]. Dietary diversity had shown no significant difference between the age of the respondents in our study, but the study done in South Africa [48] revealed younger women significantly had inadequate dietary diversity. This difference might be attributed to the methodological difference in sample selection in which the previous study was carried out in a semi-urban area selected purposively whereas the present study was based on the pregnant women living in both urban and rural areas.

In the present study, non-educated pregnant women were more likely to consume an inadequate diet compared to college and above completed pregnant women. The studies conducted in Kenya [40] and Bangladesh [49] also revealed pregnant women dietary diversity was low among those with a class of lower education. Cultural taboos accustomed by the community might have affected pregnant women to depend on less diversified food as previous research in Ethiopia [50] demonstrated practice of food taboos was common in those with no formal education.

Gravidity and parity had shown no significant association with dietary diversity during pregnancy in the study area. Similarly, a study from Pakistan [5] reported parity did not show association with dietary diversity.

This study identified that counseling on dietary diversity during ANC had a positive association with women dietary diversity. Inadequate dietary intake was significantly higher among women who did not receive dietary counseling during pregnancy. It has been proven that dietary counseling significantly increases the number of food groups consumed by pregnant women [51].

This study revealed that pregnant women in the poorest, poorer, poor and richer wealth index were more vulnerable for inadequate dietary diversity. The finding is consistent with the findings from previous studies done among lactating women in Ethiopia [52], pregnant women in Kenya [40] and India [53] that reported inadequate dietary diversity was significantly higher among women with lower income. A study done in Pakistan [5] reported dietary diversity had no significant difference based on economic status. No previous study has revealed that the poorest are good at having diversified food. Ethiopian women usually consume the most locally available, accessible and culturally acceptable foods [54, 55].

This study clarified that increasing meal frequency improves women dietary diversity. Changing the type of diet of breakfast, lunch, and dinner is commonly practiced in all parts of the country [56], and thus could be a reliable means for pregnant women to get adequate nutrients sourced from different food groups that they consume as the practice of changing food item.

## Strengths and limitations

The study findings conform to the standards of FAO and FANTA III minimum dietary diversity of women, thus the first in its kind in Amhara Regional State, which can serve as evidence for the Regional Health Bureau. Nutrition experts have judged the locally modified tool to include locally consumed food types. However, the study has not assessed the effect of seasonal variation in women’s dietary diversity score.

## Conclusion and recommendation

Consumption of less diversified food during pregnancy is typical to the study area. Adequacy of micronutrients is insufficient for more than half of the studied pregnant women. Non-educated pregnant women depend on less diversified food. Providing dietary counseling during pregnancy has been identified as an opportunity to improve nutritional practice for pregnant women. Healthcare workers can provide sustained counseling and demonstration of food preparation to help pregnant women consume more diversified food groups available in their home.

## Abbreviations

ANC: Antenatal Care
AOR: Adjusted Odds ratio
CBA: Childbearing age
CI: Confidence Interval
COR: Crude Odds ratio
DM: Debre Markos (*The name of a town)*
FANTA: Food and Nutrition Technical Assistance
FAO: Food and Agricultural Organization
HDDS: Household Dietary Diversity Score
NNP: National Nutrition Program
SD: Standard Deviation
SPSS: Statistical Package for Social Sciences
WDDS: Women Dietary Diversity Scores
WHO: World Health Organization
